# Pseudo-rheumatic manifestations of limping: Camptodactyly–arthropathy–coxa vara–pericarditis syndrome: Single case report and review of the literature

**DOI:** 10.3389/fped.2022.981938

**Published:** 2022-12-05

**Authors:** Valerio Maniscalco, Camilla Pizzetti, Edoardo Marrani, Anna Perrone, Ilaria Maccora, Ilaria Pagnini, Rosangela Artuso, Gabriele Simonini, Maria Vincenza Mastrolia

**Affiliations:** ^1^ Department of Health Sciences, University of Florence, Firenze, Italy; ^2^ Rheumatology Unit, Meyer Children's University Hospital, Firenze, Italy; ^3^ Radiology Unit, Meyer Children's University Hospital, Firenze, Italy; ^4^ Medical Genetics Unit, Meyer Children's University Hospital, Firenze, Italy; ^5^ NEUROFARBA Department, University of Florence, Rheumatology Unit, Meyer Children's University Hospital, Firenze, Italy

**Keywords:** CACP syndrome, limping in children, temporo mandibular joint, juvenile idiopathic arthritis, coxa vara

## Abstract

Camptodactyly–arthropathy–coxa vara–pericarditis (CACP) syndrome is a rare genetic disease characterized by tetrad camptodactyly, noninflammatory arthropathy, coxa vara deformity, and pericardial effusion. Arthropathy typically affects large joints and presents with joint swelling in the absence of other signs of inflammation. We described the case of a girl affected by CACP syndrome caused by a novel compound heterozygous variant in proteoglycan 4 gene (c.2831_2832insT; c.3892C > T) and associated with temporomandibular involvement. The patient received treatment with intra-articular hyaluronic acid injections, which presented rapid but transient improvements of pain and range of motion. A literature review of previously reported CACP patients has been performed. Of the patients. 69.2% (101 out of 146) were Middle Eastern, and 65.7% (96) were consanguineous. The median age of onset was 24 months (interquartile range of 12–36 months), and median age of diagnosis was 96 months (interquartile range of 48–156 months). Arthropathy was always present, mainly involving hips (95.2%), knees (92.4%), wrists (87.7%), elbows (79.5%), and ankles (57.5%). Camptodactyly and pericardial effusion were described, respectively, in 97.3% (142) and 15.1% (22) of patients. The main radiological findings were coxa vara (95.2%), femoral changes (64.4%), intraosseus cysts (14.4%), and bone erosion (5%). Of the patients, 32.9% (48) had received a previous juvenile idiopathic arthritis diagnosis. CACP syndrome can be easily misdiagnosed with juvenile idiopathic arthritis. A prolonged lack of response to immunosuppressive therapy associated with typical clinical and radiological features should prompt consideration of this rare syndrome.

## Introduction

Camptodactyly–arthropathy–coxa vara–pericarditis (CACP) syndrome is a genetic condition with autosomal recessive inheritance due to various mutations leading to the inactivation of the proteoglycan 4 (PRG4) gene on chromosome 1 (1). CACP syndrome has been found in different ethnic populations. Due to its rarity, CACP syndrome is probably underdiagnosed, making it difficult to estimate its real prevalence. PRG4 gene encodes for lubricin, a secreted glycoprotein produced by synoviocytes and chondrocytes, which lubricates the articular joints. Clinical manifestations of CACP syndrome can vary, even within families. The syndrome clinically manifests with congenital or early onset camptodactyly and noninflammatory arthropathy characterized by synovial hyperplasia and clear synovial fluid, low in cell count (2). Progressive coxa vara deformity and/or noninflammatory pericardial or pleural effusions are typical manifestations (3). CACP syndrome is commonly characterized by arthropathic involvement of large joints, such as elbows, hips, knees, and ankles (4). Typically, no temporomandibular joint (TMJ) involvement has been reported. Differential diagnosis with juvenile idiopathic arthritis (JIA) is challenging at disease onset, leading to frequent delays in the identification of the condition and prolonged ineffective therapeutic courses with immunosuppressive drugs. The absence of clinical and biochemical signs of inflammation and peculiar radiological features are the primary elements of a proper diagnosis. A prompt recognition may prevent the patient's exposure to unnecessary therapies with immunomodulatory drugs. To date, no specific treatment is available, and the therapeutic management is symptomatic.

In this regard, we report the first CACP syndrome case with TMJ arthropathy and we describe a novel compound heterozygous variant in the PRG4 gene. A literature review of previously reported CACP patients was also performed.

## Case report

**Figure 1:**
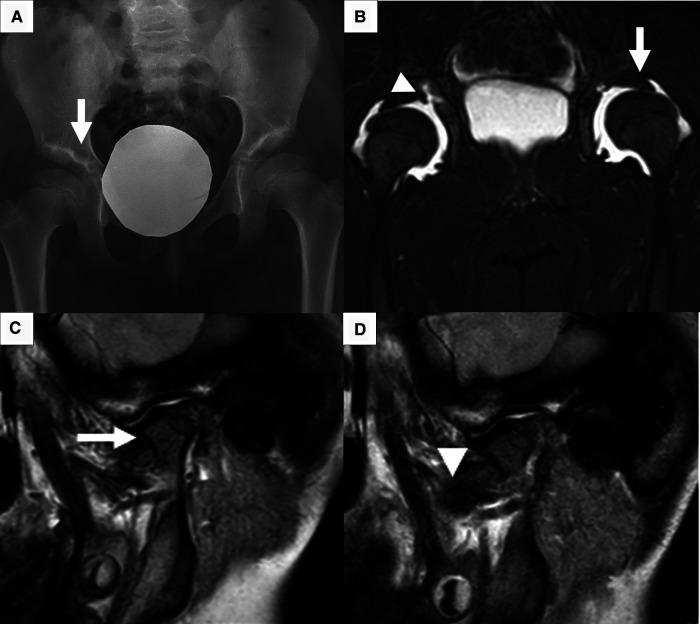
Main radiological feature. (**A**) X-ray of the pelvis shows flattening of femoral heads, stubby and short femoral neck, and bilateral coxa vara. Acetabular roofs are irregular with sclerotic surfaces and radiolucent lytic areola (*arrow*) as per acetabular cyst. (**B**) Short tau inversion recovery sequence of pelvis MRI shows effusion at coxofemoral joints resulting in partial femoral head dislocation (*arrow*); acetabular cyst connected with joint effusion (*arrowhead*). (**C, D**) MRI of left TMJ in close and open mouth shows little/no excursion of the condyle. The condyle has an enlarged and flattened morphology (*arrow*) with irregular surface. A slim layer of intra-articular effusion is present. The articular disc (*arrowhead*) has normal morphology and signal.

**Figure 2:**
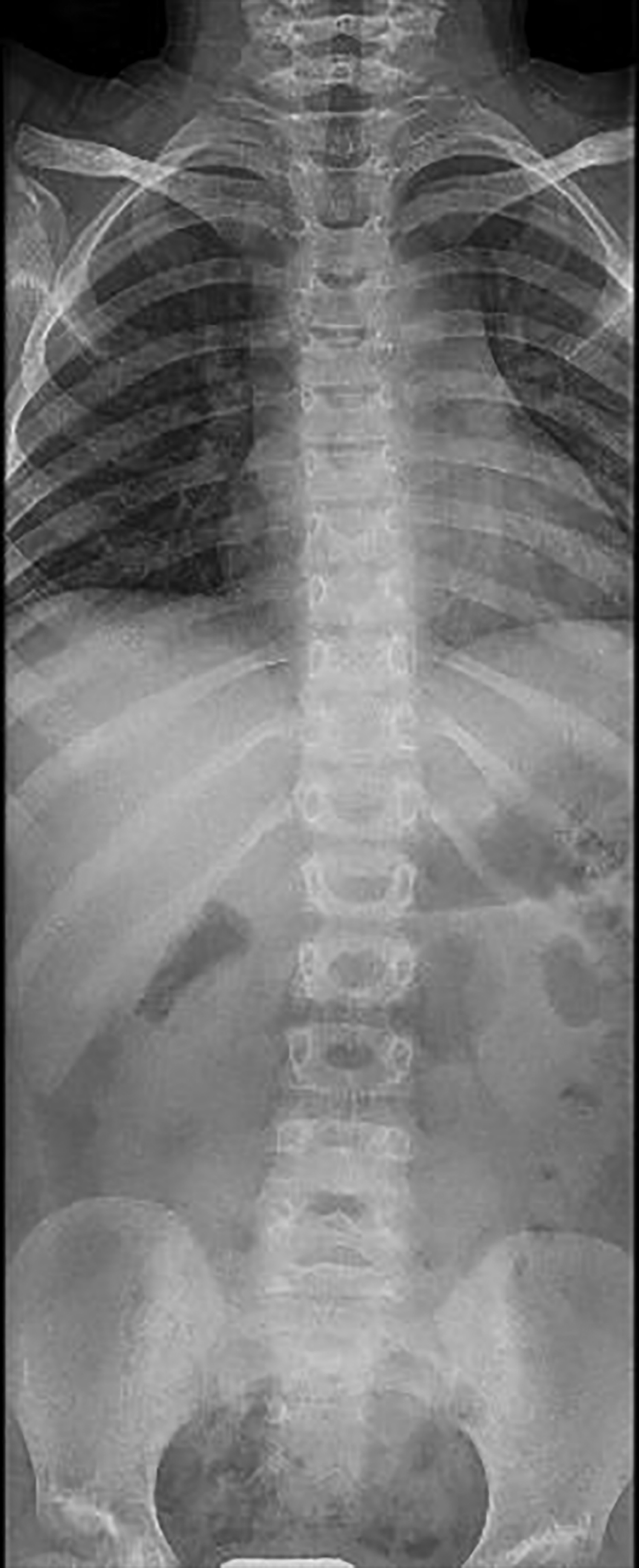
Radiography of the spine. X-ray of the spine shows on the coronal plane the presence of wide-radius convex left curvature of the thoracolumbar vertebrae.

A 4-year-old girl was referred to the Rheumatology Unit of Meyer Children's University Hospital, Florence, in June 2015 because of asthenia, knee pain following trivial effort, and an unsteady gait since she started walking at 18 months of age. She was the first child of nonconsanguineous Caucasian parents with no relevant family history, except for autoimmune thyroiditis in the mother. Past medical history was unremarkable. Physical examination revealed bilateral arthritis of the ankles, knees, metacarpophalangeal and proximal interphalangeal joints, camptodactyly, and genu valgus. Laboratory tests displayed normal values of blood count, erythrocyte sedimentation rate (ESR), C-reactive protein (CRP), and negativity of antinuclear antibodies (ANA). The girl, diagnosed with polyarticular JIA, started subcutaneous methotrexate (15 mg/m^2^/week) and underwent intra-articular steroid injections in ankles and knees. She continued the multidisciplinary follow-up with clinical, laboratory, and instrumental evaluations. The patient never developed uveitis in serial ophthalmologic examinations, and inflammatory laboratory markers were always negative. During the evaluations, radiological scans of different skeletal segments were performed. Pelvis and lower limbs x-ray in weight-bearing position (October 2016) showed coxa vara and stubby and short femoral necks; and wide acetabulum with joint space enlargement, acetabular cyst, and signs of erosion (Figure 1). Spine radiography (November 2016) reported a mild reduction in lumbar lordosis and left convex thoracolumbar scoliosis (Figure 2). Bone densitometry (October 2016) revealed normal bone mineral density values. Pelvis and knee magnetic resonance imaging (MRI) (December 2016) identified synovial hyperplasia accompanied by joint effusion and bone erosions in both the coxofemoral joints and knees (Figure 1). Ankle-foot MRI (December 2016) showed bilateral effusion in the tibiotarsal joints.

Due to persistent disease activity, the girl underwent several intra-articular steroid injections and oral corticosteroid courses. In addition, different treatments with biological drugs were attempted but proved ineffective. In December 2015, the patient started etanercept; then, due to lack of efficacy, on April 2016, etanercept was replaced by abatacept together with an oral course of corticosteroid (August 2016–September 2016). In January 2017, because of the occurrence of pseudotumor cerebri, methotrexate was interrupted. In February 2017, after abatacept discontinuance, tocilizumab was started along with an oral steroid course until October 2017 without a beneficial response. Subsequently, a partial improvement was only observed with oral and intra-articular steroid administration.

Due to her peculiar clinical features, with no disease remission and no response to treatment, trios whole exome sequencing (WES) was performed in September 2018. All identified variants have been classified as potentially pathogenic or benign variants in accordance with the interpretation guidelines of the American College of Medical Genetics (ACMG) (5). The analysis revealed a compound heterozygous mutation in the PRG4 gene (c.2831_2832insT; c.3892C > T); both mutations were point nonsense-mutations not previously reported in the literature. The first mutation c.2830_2831insT, inherited from the mother, caused the insertion of one thymine nucleotide in exon 7, producing a premature truncation of the protein's 34 amino acids downstream to codon 944 (*p*.Thr944IlefsTer34). The second variant c.3892C > T, inherited from the father, caused the substitution of glutamic acid in position 1,298 with a stop codon in exon 11. According to ACMG guidelines, both variants were classified as likely pathogenic. To date, stop mutations and small insertions or deletions in PRG4 gene are described in the literature as associated with CACP syndrome (OMIM#208250). Our patient has never experienced pericarditis, but considering these genetic results, a cardiologic evaluation was performed resulting normal. Since the diagnosis (June 2019), the patient received monthly intra-articular injections of hyaluronic acid in knees and ankles and started a tailored rehabilitative program with a team of rheumatologists, physiatrists, and physiotherapists. The girl referred reduced pain soon after every infiltration with an improvement in the range of motion. However, this effect was transient since the pain returned after 3 weeks from each hyaluronic acid injection. In September 2019, the patient reported a left-sided TMJ pain associated with an impairment in the opening of the mouth. TMJ MRI revealed bilateral anterior dislocation of the articular disc more evident on the left side, irregular and flattened condyle articular surface with erosive images on the posterior face (Figure 1). Slim intra-articular fluid and thickened posterior tissues were seen on the left side. After orthodontic evaluation, a tailored appliance was later pursued with prompt reduction of mandibular pain.

Currently, the girl continues her multidisciplinary follow-up through an integrated physiatric, physiotherapic, and rheumatologic management. Pain and reduced range of motion had improved but persisted. No adverse events were reported.

## Discussion

CACP syndrome is a rare autosomal recessive condition caused by the mutation of the PRG4 gene. The syndrome was first described in 1986 by Bulutlar et al. and is characterized by tetrad camptodactyly, noninflammatory arthropathy, coxa vara deformity, and pericardial effusion (6). The arthropathy typically starts during childhood and involves large joints, such as knees, wrists, elbows, ankles, and hips, while shoulder and spine involvement are described sporadically (7, 8)*.* We reported the first case of temporomandibular involvement in CACP syndrome. A literature search was carried out using PubMed/Medline and Embase databases. English language reports of CACP syndrome reported from 1986 until 1 April 2022 were retrieved and analyzed. Twenty-eight studies (12 case series, 11 case reports, 5 retrospective multicenter cohort studies) had previously described CACP patients, recording a total of 146 patients, 94 males and 52 females (Table 1) (2–4, 6–30). Double-reported patients were excluded from the total account (2, 11, 15).

**Table 1:** CACP syndrome patients reported till date.

Publication	Country	Number of patients (M/F)	Consanguinity	Mutations reported	Age of diagnosis (months)	Clinical features				Therapy
						*Camptodactyly*	*Large joint arthropathy*	*Coxa vara*	*Pericarditis*	
Bulutlar 1986	Turkey	4 (0/4)	4	NA	7–16	3	4	4	2	NA
Laxer 1986	Canada	1 (1/0)	1	NA	60	1	1	0	1	NSAID, intra-articular steroid, physiotherapy
Bahabri 1994	Saudi Arabia	3 (1/2)	0	NA	84–156	3	3	3	0	Oral gold, physiotherapy
Bahabri 1998^a^	Saudi Arabia	8 (5/3)	1	NA	24–156	8	8	7	2	Surgery (femoral osteotomy, repair of hallux valgus deformity)
Faivre 2000	Europe, North Africa	12 (10/2)	8	NA	24–180	12	12	10	0	Surgery (6 patients)
El-Garf 2003	Egypt	10 (7/3)	10	NA	36–204	10	10	9	0	NA
Choi 2004	Korea	1 (1/0)	0	NA	120	1	1	0	1	NA
Offiah 2005	United Kingdom	1 (1/0)	0	NA	156	1	1	1	1	NSAID, methotrexate, systemic steroids, anti-TNF
Alazami 2006	Saudi Arabia	7 (5/2)	4	c.3139_3140delAA c.4078A > T c.923_924delAA c.3125_3128delGAGT c.3276_3277delAA	18–60	7	7	5	0	NA
Al-Mayouf 2007^a^	Saudi Arabia	16 (11/5)	16	NA	44	16	16	14	2	NA
Basit 2011	Pakistan	9 (8/1)	9	c.2816_2817delAA	NA	9	9	9	0	NA
Akawi 2012	United Arab Emirates	4 (2/2)	4	c.1320dupC	60–144	4	4	4	0	Surgery (correction of flexion contracture)
Murphy 2012	United States	2 (1/1)	0	NA	168–204	2	2	2	1	Intra-articular steroid, surgery (hip arthroplasty)
Albuhairan 2013^a^	United Arab Emirates	22 (15/7)	16	NA	12–168	22	22	22	2	NSAID, methotrexate (10 patients), systemic steroid
Emad 2013	Egypt	1 (0/1)	0	NA	96	1	1	1	1	NA
Kakkar 2013	India	1 (0/1)	1	NA	39	1	1	1	1	NA
Ciullini Mannurita 2014	Italy, Albania, Netherlands	13 (8/5)	0	c.2754_2758delGACAA c.3636þ 3A4G c.3894_3898delGGTTA c.1982_1983delCT c.3648C4A c.2153delA	1–180	10	13	11	4	NA
Nandagopalan 2014	India	3 (2/1)	0	c.2883_2886delAAGA c.2883_2886delAAGA 2645_2646delGA	60–192	3	3	0	0	NA
Tasar 2014	Turkey	1 (1/0)	0	NA	84	1	1	1	1	Physiotherapy, surgery
Madhusudan 2016	India	2 (2/0)	0	NA	48–96	2	1	2	0	NSAID, physiotherapy
Patil 2016	India	2 (2/0)	0	NA	108–156	2	2	1	2	NA
Peters 2016	Netherlands	1 (0/1)	1	c.1290de	120	1	1	1	1	Pericardiectomy, cotrimoxazole prophylaxis, immunoglobulin supplement iv
Vutukuru 2016	India	1 (1/09)	0	NA	96	0	1	1	0	NA
Yilmaz 2017	Turkey	35 (20/15)	28	c.1194delC c.73934del c.3276_3277delAA c.4101C > G c.3276_3277delAA c.1192delC c.1911delT c.2215A > T c.1910_1911delCT c.2837_2838delAA c.849delA c.4101C > G	12–624	35	35	35	3	Surgery
Al-Mayouf 2017^a^	United Arab Emirates	6 (3/3)	NA	NA	NA	NA	6	NA	NA	Medical synovectomy (radiosynovectomy) using radioactive isotope with no benefit
Albtoush 2018	Jordan	1 (0/1)	0	NA	132	1	1	1	1	Surgery (tendon-lengthening of fingers)
Kisla Ekinci 2021	Turkey	3 (0/3)	3	c.538C > T, c.1194delC, c.1194del	36–156	3	3	1	0	NSAID, methotrexate, anti-TNF, physiotherapy, surgery
Johnson 2021	India	2 (1/1)	1	c186275834dupT *p*.Ser1085Ter	144–156	2	2	2	2	NA

NSAID, nonsteroidal anti-inflammatory drugs; anti-TNF: anti-tumor necrosis factor; NA: not available.

^a^ Studies that included patients who have been already reported.

CACP syndrome is a rare clinical condition, and its real prevalence is still unknown. Our patient was a Caucasian girl with nonconsanguineous parents. However, this genetic disorder has been observed in patients of different ethnicities, especially in Middle Eastern families, and is frequently associated with consanguinity. As regards the previously reported cases, 69.2% (101) of patients were of Middle Eastern descents (41 from Saudi Arabia, 39 from Turkey, 16 from north Africa, 4 from the United Arab Emirates, and 1 from Jordan), 16.4% (24) were Caucasian (10 from Italy, 4 from France, 2 from Albany, 2 from Netherlands, 2 from England, 2 from United States, 1 from Ireland, and 1 from Switzerland), 13.7% (20) were from south Asia (11 from India, 9 from Pakistan), and 1 from east Asia (Korea) (2–4, 6–30). Consanguinity was reported in 65.7% (96) of CACP cases (2, 3, 15–17, 23, 25, 27–29).

The articular involvement first appears in early childhood, leading to an incomplete clinical picture. Moreover, polyarticular arthropathy can mimic JIA, often leading to a delayed diagnosis and inappropriate treatments.

Our patient presented articular symptoms at 18 months of age, received a mistaken diagnosis of polyarticular JIA at 4 years of age and received a final diagnosis of CACP when she was 8 years old. The median age of symptom onset in CACP patients is 24 months (interquartile range, IQR, 12–36 months) with a median age of diagnosis of 96 months (IQR 48–156 months); one-third of them (32.9%, 48 patients) were diagnosed as JIA (2–4, 6–30).

Our girl presented camptodactyly, hip, knee, and ankle arthropathy complaining pain after trivial efforts. In addition, subsequent temporomandibular involvement was observed. Polyarticular arthropathy represents a constant finding in CACP syndrome, mainly affecting the large joints bilaterally, the hips (95.2%), knees (92.4%), wrists (87.7%), elbows (79.5%), and ankles (57.5%). Patients typically present joint swelling without any other sign of inflammation. Pain was reported in only one-fifth of patients (35) and 37% (54) presented a limited range of motion. Camptodactyly, usually bilateral and congenital, affected 97.3% (142) of patients, although it may appear in the first months of life (2–4, 6–30). Pericardial and pleural effusions were described in 15.1% (22) and 4.8% (7) of cases, respectively (2, 3, 8, 9, 12, 13, 15, 18, 19, 22, 23, 25, 27, 30). Other associated conditions have been reported, although they are certainly not related to this syndrome. Two patients presented hepatomegaly and ascites, and two had constrictive pericarditis complicated by portal hypertension and protein-losing enteropathy in one case (12, 23, 25, 27, 30). Congenital cataract was observed in one patient (17).

As with our girl, all CACP patients had normal inflammatory marker values (i.e., ESR and CRP) in contrast to polyarticular JIA children, in which they are typically elevated.

Concerning the radiological features, our case presented characteristic CACP findings: the pelvic x-ray showed coxa vara deformity associated with femoral changes (stubby and short femoral necks) and increased joint space secondary to joint effusion. The pelvis and knee MRI reported synovial hyperplasia with joint effusion and bone erosions. An MRI of the TMJ showed intra-articular fluid associated with signs of bone erosion and thickening of periarticular soft tissue. Moreover, the patient presented with left convex thoracolumbar scoliosis.

In this regard, coxa vara deformity was almost always present in CACP patients (95.2%) associated with femoral changes (e.g., short femoral neck, flat and irregular femoral head) in more than half of cases (64.4%) (2–4, 7, 8, 13, 16–18, 21, 22, 24, 25, 27). Bone cysts, secondary to joint capsule herniation, are characteristic of the syndrome and occasionally reported (14.4%) (2, 4, 7, 9, 13, 21, 24, 25, 28, 29). Osteoporosis was observed in 36.3% of cases (53), while spine abnormalities (e.g. kyphosis, lordosis, scoliosis) were described in 24.7% of cases (36) (2–4, 12, 16, 20, 23, 25, 27). Signs of bone erosion are rarely described (5% of cases), as in our patient (7, 19). Conventional radiographs also revealed periarticular soft tissue swelling and osteopenia (15). Imaging findings of articular effusion and synovial hyperplasia were reported in 40.4% patients (59) at ultrasound sonography or MRI (2, 4, 7–9, 12, 13, 17, 19, 20, 24, 28, 29).

The synovial tissue histology, performed in 20 patients, revealed little or no mononuclear infiltration with typical multinucleated giant cells (CD68+) infiltration. Synovial fluid was always noninflammatory (clear, honey-colored, and low in cell count) (3, 4, 9, 10, 12, 13, 22, 23, 28).

The patients received several immunosuppressive therapies, including biologic agents, without any significant improvement, and multiple systemic and intra-articular corticosteroid courses with only transient relief. Indeed, CACP syndrome typically shows no response to immunomodulatory treatment with only symptomatic therapy. In some cases (eight patients), physiotherapy allowed a recovery of range of motion and a reduction in joint pain (9, 21, 24, 29).

Because CACP syndrome is caused by the mutation of the PRG4 gene that encodes for lubricin, a proteoglycan secreted by synoviocytes and chondrocytes acts as a lubricant in joint space and protects cartilage surfaces (31). Hyaluronic acid, similar to lubricin, may supply joint lubrication and protect the cartilage from mechanical degradation (32). Intra-articular hyaluronic acid is used in the elderly with osteoarthritis and has been shown to have beneficial effect on pain and joint function without presenting safety risk (32–34)*.* Starting from these considerations, we decide to treat our patient with intra-articular hyaluronic acid injections with partial benefit. The girl presented with pain reduction soon after each infiltration and an improvement in range of motion, although with a transient effect of 3 weeks. A surgical approach has been attempted with variable effects in 29 CACP cases (19.9%). Four patients underwent surgical corrections of flexion contractures of fingers with success, and two patients underwent hip arthroplasty with pain relief and improved joint function (17, 18). Six children underwent radiosynovectomy with no benefit (26). Finally, one patient underwent right femoral osteotomy and surgical repair of hallux valgus deformity, and two patients underwent pericardiectomy (9, 23, 28). In 14 cases, the type of surgical intervention was not reported (3, 25, 29).

In conclusion, CACP syndrome is a rare disorder that can be incorrectly framed as JIA causing a delay in diagnosis and inappropriate treatment with immunosuppressive drugs including biologic agents. The association with camptodactyly and coxa vara (almost always present), the involvement of large joints, and the absence of clinical and biochemical inflammatory signs are hallmarks of the syndrome. These findings associated with a prolonged lack of response to immunosuppressive therapy should induce the physician to reconsider an earlier JIA diagnosis. Typical radiological features identified by x-ray and ultrasonography can support the diagnosis. Synovial pathology may be helpful even if not mandatory. Genetic testing definitively confirms the diagnosis and allows for genetic counseling for the family members. Currently, no specific treatment is available, and therapeutic management is only symptomatic and requires a multidisciplinary approach.

## Data Availability

The original contributions presented in the study are included in the article/Supplementary Material, further inquiries can be directed to the corresponding author.

## References

[1] Marcelino J Carpten JD Suwairi WM Gutierrez OM Schwartz S Robbins C CACP, encoding a secreted proteoglycan, is mutated in camptodactyly-arthropathy-coxa vara-pericarditis syndrome. Nat Genet. (1999) 23:319–22. 10.1038/15496 10545950

[2] Albuhairan I Al-Mayouf SM. Camptodactyly-arthropathy-coxavara-pericarditis syndrome in Saudi Arabia: clinical and molecular genetic findings in 22 patients. Semin Arthritis Rheum. (2013) 43:292–6. 10.1016/j.semarthrit.2012.11.004 23290693

[3] Faivre L Prieur A-M Le Merrer M Hayem F Penet C Woo P Clinical variability and genetic homogeneity of the camptodactyly-arthropathy-coxa vara-pericarditis syndrome. Am J Med Genet. (2000) 95:233–6. 10.1002/1096-8628(20001127)95:3233:aid-ajmg9>3.0.co;2-3 11102929

[4] Kakkar RM Soneji S Badhe RR Desai SB. Camptodactyly-arthropathy-coxa vara-pericarditis syndrome: important differential for juvenile idiopathic arthritis. J Clin Imaging Sci. (2013) 3:24. 10.4103/2156-7514.114211 24083061 PMC3779395

[5] Richards S Aziz N Bale S Bick D Das S Gastier-Foster J Standards and guidelines for the interpretation of sequence variants: a joint consensus recommendation of the American college of medical genetics and genomics and the association for molecular pathology. Genet Med. (2015) 17:405–24. 10.1038/gim.2015.30 25741868 PMC4544753

[6] Bulutlar G Yazici H Ozdoğan H Schreuder I. A familial syndrome of pericarditis, arthritis, camptodactyly, and coxa vara. Arthritis Rheum. (1986) 29:436–8. 10.1002/art.1780290321 3964320

[7] Albtoush OM Taib AA Manzalawi KA Mahafza WS. Camptodactyly-arthropathy-coxa vara-pericarditis syndrome with shoulder joint involvement: a case report with literature review. Rofo. (2018) 190:856–8. 10.1055/s-0043-120765 29156476

[8] Emad Y Ragab Y Khalifa M Bassyouni I El-Shaarawy N Rasker JJ. Axial involvement with facet joint arthropathy and bony ankylosis in a case of camptodactyly, arthropathy, coxa vara, pericarditis (CACP) syndrome. Joint Bone Spine. (2013) 80:520–2. 10.1016/j.jbspin.2013.01.010 23931850

[9] Bahabri S Sakati N Hugosson C Hainau B Al-Balla SR Al-Mazyed A Syndrome of camptodactyly, arthropathy and coxa vara (CAC syndrome). Ann Saudi Med. (1994) 14:479–82. 10.5144/0256-4947.1994.479 17587953

[10] Laxer RM Cameron BJ Chaisson D Smith CR Stein LD. The camptodactyly-arthropathy-pericarditis syndrome: case report and literature review. Arthritis Rheum. (1986) 29:439–44. 10.1002/art.1780290322 3964321

[11] Bahabri SA Suwairi WM Laxer RM Polinkovsky A Dalaan AA Warman ML. The camptodactyly-arthropathy-coxa vara-pericarditis syndrome: clinical features and genetic mapping to human chromosome 1. Arthritis Rheum. (1998) 41:730–5. 10.1002/1529-0131(199804)41:4730:AID-ART22>3.0.CO;2-Y 9550484

[12] Choi BR Lim YH Joo KB Paik SS Kim NS Lee JK Camptodactyly, arthropathy, coxa vara, pericarditis (CACP) syndrome: a case report. J Korean Med Sci. (2004) 19(6):907–10. 10.3346/jkms.2004.19.6.907 15608409 PMC2816297

[13] Offiah AC Woo P Prieur AM Hasson N Hall CM. Camptodactyly-arthropathy-coxa vara-pericarditis syndrome versus juvenile idiopathic arthropathy. AJR Am J Roentgenol. (2005) 185(2):522–9. 10.2214/ajr.185.2.01850522 16037531

[14] Alazami AM Al-Mayouf SM Wyngaard C-A Meyer B. Novel PRG4 mutations underlie CACP in Saudi families. Hum Mutat. (2006) 27:213. 10.1002/humu.9399 16429407

[15] Al-Mayouf SM. Familial arthropathy in Saudi Arabian children: demographic, clinical, and biochemical features. Semin Arthritis Rheum. (2007) 36:256–61. 10.1016/j.semarthrit.2006.08.008 16996580

[16] Basit S Iqbal Z Umicevic-Mirkov M Kamran Ul-Hassan Naqvi S Coenen M. A novel deletion mutation in proteoglycan-4 underlies camptodactyly-arthropathy-coxa-vara-pericarditis syndrome in a consanguineous Pakistani family. Arch Med Res. (2011) 42:110–4. 10.1016/j.arcmed.2011.04.006 21565623

[17] Akawi NA Ali BR Al-Gazali L. A novel mutation in PRG4 gene underlying camptodactyly-arthropathy-coxa vara-pericarditis syndrome with the possible expansion of the phenotype to include congenital cataract. Birth Defects Res A Clin Mol Teratol. (2012) 94:553–6. 10.1002/bdra.23031 22678705

[18] Murphy JM Vanderhave KL Urquhart AG. Total hip arthroplasty in adolescents with severe hip arthropathy and dysplasia associated with camptodactyly-arthropathy-coxa vara-pericarditis syndrome. J Arthroplasty. (2012) 27:1581.e5–8. 10.1016/j.arth.2012.01.007 22386609

[19] Ciullini Mannurita S Vignoli M Bianchi L Kondi A Gerloni V Breda L CACP syndrome: identification of five novel mutations and of the first case of UPD in the largest European cohort. Eur J Hum Genet. (2014) 22:197–201. 10.1038/ejhg.2013.123 23756439 PMC3895642

[20] Nandagopalan RS Phadke SR Dalal AB Ranganath P. Novel mutations in PRG4 gene in two Indian families with camptodactyly-arthropathy-coxa vara-pericarditis (CACP) syndrome. Indian J Med Res. (2014) 140(2):221–6. PMID: 25297354; PMCID: PMC4216495 25297354 PMC4216495

[21] Madhusudan S Gupta A Prakash M Matta D Suri D Singh S. Camptodactyly-arthropathy-coxa vara-pericarditis (CACP) syndrome: a mimicker of juvenile idiopathic arthritis. Scand J Rheumatol. (2016) 45:77–8. 10.3109/03009742.2015.1085085 26508154

[22] Patil DV Phadke MS Pahwa JS Dalal AB. Brothers with constrictive pericarditis—a novel mutation in a rare disease. Indian Heart J. (2016) 68(Suppl 2):S284–7. 10.1016/j.ihj.2016.03.020 27751317 PMC5067734

[23] Peters B Schuurs-Hoeijmakers JHM Fuijkschot J Reimer A van der Flier M Lugtenberg D Protein-losing enteropathy in camptodactyly-arthropathy-coxa vara-pericarditis (CACP) syndrome. Pediatr Rheumatol Online J. (2016) 14:32. 10.1186/s12969-016-0093-5 27224999 PMC4880819

[24] Vutukuru R Reddy KKM. Pathognomonic acetabular cysts in camptodactyly-arthropathy-coxa vara-pericarditis (CACP) syndrome. Indian J Med Res. (2016) 143:834–5. 10.4103/0971-5916.192082 27748313 PMC5094128

[25] Yilmaz S Uludağ Alkaya D Kasapçopur Ö Barut K Akdemir ES Celen C Genotype-phenotype investigation of 35 patients from 11 unrelated families with camptodactyly-arthropathy-coxa vara-pericarditis (CACP) syndrome. Mol Genet Genomic Med. (2018) 6:230–48. 10.1002/mgg3.364 29397575 PMC5902402

[26] Al-Mayouf SM Almutairi N Alismail K. The efficacy of Yttrium-90 radiosynovectomy in patients with camptodactyly-arthropathy-coxa vara-pericarditis syndrome. Mol Imaging Radionucl Ther. (2017) 26:33–7. 10.4274/mirt.29484 28291008 PMC5350503

[27] Johnson N Chaudhary H Kumrah R Pilania RK Sharma Y Sharma A Syndrome of progressive deforming non-inflammatory arthritis of childhood: two patients of camptodactyly-arthropathy-coxa vara-pericarditis syndrome. Rheumatol Int. (2021) 41:1875–82. 10.1007/s00296-020-04688-0 32813152

[28] Kisla Ekinc RM Balci RM Dogan H Ceylaner S Varan C Erdem S Camptodactyly-arthropathy-coxa vara-pericarditis syndrome resembling juvenile idiopathic arthritis: a single-center experience from Southern Turkey. Mol Syndromol. (2021) 12:112–7. 10.1159/000513111 34012381 PMC8114071

[29] Taşar M Eyileten Z Kasýmzade F Uçar T Kendirli T Uysalel A. Camptodactyly-arthropathy-coxa vara-pericarditis (CACP) syndrome. Turk J Pediatr. (2014) 56:694–9. 10.1002/art.1780290321 26388606

[30] El-Garf A Mahmoud G Gheith R Abd El-Aaty G Abd El-Aaty H. Camptodactyly, arthropathy, coxa vara, and pericarditis syndrome among egyptians. J Rheumatol. (2003) 30:1081–6. 10.1002/art.1780290321 12734910

[31] Rhee DK Marcelino J Baker M Gong Y Smits P Lefebvre V The secreted glycoprotein lubricin protects cartilage surfaces and inhibits synovial cell overgrowth. J Clin Invest. (2005) 115:622–31. 10.1172/JCI22263 15719068 PMC548698

[32] Altman RD Bedi A Karlsson J Sancheti P Schemitsch E. Product differences in intra-articular hyaluronic acids for osteoarthritis of the knee. Am J Sports Med. (2016) 44:2158–65. 10.1177/0363546515609599 26578719

[33] He W-W Kuang M-J Zhao J Sun L Lu B Wang Y Efficacy and safety of intraarticular hyaluronic acid and corticosteroid for knee osteoarthritis: a meta-analysis. Int J Surg. (2017) 39:95–103. 10.1016/j.ijsu.2017.01.087 28137554

[34] Altman R Hackel J Niazi F Shaw P Nicholls M. Efficacy and safety of repeated courses of hyaluronic acid injections for knee osteoarthritis: a systematic review. Semin Arthritis Rheum. (2018) 48:168–75. 10.1016/j.semarthrit.2018.01.009 29496227

